# Antenna Effect in Halogen‐Containing ZnSm Coordination Compounds: Utilizing Colorimetry for a Room‐Temperature Tunable Ratiometric Molecular Thermometer

**DOI:** 10.1002/advs.75323

**Published:** 2026-04-20

**Authors:** Ye Xia, Yingshan Xue, Feng Pan, Ai Wang, Hong Huang, Zhenxing Li, Bing‐Wu Wang, Song Gao

**Affiliations:** ^1^ Spin‐X Institute School of Chemistry and Chemical Engineering South China University of Technology Guangzhou P. R. China; ^2^ Beijing National Laboratory for Molecular Sciences College of Chemistry and Molecular Engineering Peking University Beijing P. R. China; ^3^ State Key Laboratory of Heavy Oil Processing College of New Energy and Materials China University of Petroleum (Beijing) Beijing P. R. China; ^4^ Key Laboratory of Chemical Biology and Molecular Engineering of the Education Ministry Institute of Molecular Science Shanxi University Taiyuan P. R. China; ^5^ Guangdong Basic Research Center of Excellence for Functional Molecular Engineering School of Chemistry Sun Yat‐sen University Guangzhou P. R. China

**Keywords:** dinuclear complex, halogen substitution, molecular ratiometric thermometer, photoluminescence, rare earth

## Abstract

Temperature‐dependent photoluminescence (TDPL) is an efficient optical characterization method. Compared to traditional ratiometric photothermal instruments that rely on doped materials and suffer from poor reproducibility, molecular ratiometric thermometers offer advantages such as high precision, fast response, remote temperature measurement capability, and good optical stability. We synthesized three halogen‐substituted bimetallic coordination compounds using a one‐pot method, which showed good reproducibility. The molecular complexs are abbreviated as **ZnSm‐X** (X = Cl, Br, I). Importantly, **ZnSm‐X** with different halogen substitutions exhibit different temperature dependencies at 253–333 K. Among them, the **ZnSm‐Cl** has a relative sensitivity of 12.14% K^−1^ at 268 K. This is attributed to the well‐matched relative energy levels between ligand and Sm^3+^, which facilitate energy transfer from the triplet state of the ligand to the excited state of Sm^3+^, resulting in temperature‐dependent photoluminescence. By using colorimetry, the temperature‐dependent photoluminescence of **ZnSm‐Cl** complexes within a room‐temperature adjustable range will provide new ideas for molecular ratiometric thermometers.

## Graphical abstract

The **ZnSm‐Cl** dinuclear molecular complex was synthesized through a simple one‐pot method. **ZnL** and Sm^3+^ exhibit complementary temperature‐dependent emission As the temperature increases, a noticeable color difference of **ZnSm‐Cl** can be observed within the room temperature range of 253‐333 K. **ZnSm‐Cl** exhibits a high sensitivity of 12.14% K^−1^ at 268 K, making it an ideal choice for molecular‐scale thermometers.

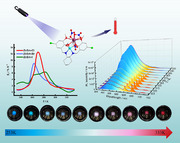

## 1 Introduction

Photoluminescence (PL) exhibits sensitive and reversible responses to external stimuli, holding great potential in various applications [1]. which include temperature sensors [2], biological probes [3], drug delivery carriers [4], and chemical sensors [5]. Temperature‐dependent photoluminescence (TDPL), due to its non‐invasive nature [6], is commonly employed in luminescent physical sensors for temperature monitoring and has demonstrated significant potential in aerospace, environmental, and industrial applications [7]. Compared to traditional thermometers, TDPL‐based luminescent sensors have notable advantages in rapid response, high sensitivity, and resistance to extreme atmospheric conditions [8]. Compared with conventional liquid‐in‐glass and bimetallic thermometers [9, 10], molecular luminescent thermometers offer distinct advantages such as high spatial resolution and sensitivity, fast response, non‐invasiveness, and inertness to strong electric or magnetic fields. However, in most reports, the temperature response is primarily associated with changes in luminescence intensity and the lifetime of a single emission transition [11]. Ratiometric thermometers based on dual emission provide self‐calibrated temperature readings. They are unaffected by concentration or fluctuations in the excitation signal, making them more reliable and accurate than thermometers based on single‐transition intensity [12]. Halogen bonds have become one of the most interesting types of non‐covalent interactions in recent years [13]. As is well known, replacing hydrogen atoms in high‐energy vibrating bonds with heavy atoms, such as in C─Cl or C─Br bonds, is a common method. This approach can effectively reduce vibrational energy, thereby minimizing energy loss caused by ligand or solvent vibrations and enhancing the effective luminescence quantum yield [14]. Riley and colleagues found that atoms or chemical groups substituted near the halogen significantly influence the strength of the halogen bond [15]. Santos and colleagues discovered that introducing halogen atoms into luminescent complexes can modulate their luminescent properties [16]. Therefore, halogenated complexes may enable significant performance improvements.

Rare‐earth‐based temperature‐sensing technology has attracted significant attention due to the excellent luminescent properties associated with the shielded nature of the 4*f* orbitals [17]. Lanthanide complex luminophores possess inherent features, including narrow emission peaks, good monochromaticity, large Stokes shifts, long excited‐state lifetimes, and strong light absorption capabilities via the antenna effect of organic ligands [18]. Their prominent advantages include long lifetimes, which enable time‐gated experiments to suppress background fluorescence [19]. Currently, doping is the most common and mature method for introducing rare‐earth ions into materials. The goal is to uniformly incorporate rare‐earth ions into stable host matrices, utilize energy transfer channels within the host to enhance luminescence efficiency, and control ion concentrations to prevent concentration quenching [20]. In the near‐infrared region, for ions such as Er, Ho, Tm, Nd, and Dy, the relative populations of two closely spaced thermally coupled levels associated with a single emission center remain in a quasi‐equilibrium state, following the Boltzmann distribution [21]. Notably, the ^2^H_11/2_ and ^4^S_3/2_ levels of the Er^3+^, with an appropriate energy gap of about 800 cm^−1^, are the most preferred thermally coupled levels. In the visible region, achieving ratiometric luminescence typically requires doping a second rare‐earth ion or an organic dye into the rare‐earth metal‐organic framework. The most commonly used system is Tb–Eu co‐doping [22]. However, due to cross‐relaxation, the ratio of doped ions must be strictly controlled. It is highly challenging to achieve identical concentrations and consistent quality of doped rare‐earth ions across different preparation batches. The distances between donor and acceptor ions are random and can only be estimated statistically. In the rare‐earth ion co‐doped system, donor‐to‐donor and acceptor‐to‐acceptor energy transfer can also occur, further complicating the transfer kinetics. To overcome the limitations of co‐doped systems, dual‐emitter molecular materials have emerged as a promising alternative [23]. Compared with rare‐earth‐doped nanomaterials, bimetallic complexes possess well‐defined structures, stable stoichiometry, and fixed donor–acceptor distances. These characteristics enable direct determination of energy transfer rates and ensure reproducible material performance, offering significant advantages for constructing ratiometric optical thermometers. Under 365 nm irradiation, organic ligands act as antennas through the energy‐level matching mechanism of the antenna effect. They absorb ultraviolet excitation energy and transfer it to rare‐earth ions, enabling energy transfer from ligands to rare earth ions and resulting in intense luminescence [24].

Here, we synthesized molecular materials with dual emission centers: Sm^3+^ and Zn^2+^‐halogen Schiff base complexes in a non‐centrosymmetric crystal system, the complexes are [1*R*,2*R*‐ZnLSm(CH_3_OH)(NO_3_)_3_] (denoted as **ZnSm‐X,  X  = Cl, Br, I,** **H**_**2**_ **L** = (N, N'‐bis(3‐methoxy‐5‐X‐salicylidene)cyclohexane‐1,2‐diamine)). This material can serve as a ratiometric molecular temperature sensor. Variable‐temperature emission spectroscopy reveals that the molecule exhibits highly sensitive, temperature‐dependent photoluminescence tunable within an 80 K range (253–333 K) at room temperature. The modulation of its photophysical properties is rationalized through the antenna effect, elucidating the temperature‐dependent photoluminescence mechanism of the complex. Emission spectrum revealed that the Schiff base ligand coordinated with Zn^2+^(forming **ZnL**) emits indigo fluorescence, with an emission peak around 470 nm. Sm^3+^ emits orange‐red fluorescence, with characteristic emission peaks at 561 nm, 599 nm, 644 nm, and 710 nm, arising from the ^4^G_5/2_ → ^6^H_5/2_, ^6^H_7/2_, ^6^H_9/2_, and ^6^H_11/2_ transitions. The emissions of the two components vary complementarily with temperature: at low temperatures, **ZnL** emission dominates, whereas at higher temperatures, Sm^3+^ emission prevails, enabling colorimetric temperature monitoring with high sensitivity, reversibility, and distinct color changes. This temperature dependence is attributed to thermally induced energy transfer facilitated by energy‐level matching. Different halogen‐substituted complexes exhibit distinct responses, with the chloro‐substituted complex showing the highest relative sensitivity of 12.14% K^−1^ at 268 K, demonstrating the potential for constructing ratiometric molecular thermometers. Current research on ratiometric molecular complexes for temperature sensing remains relatively limited. Several representative examples are summarized in Table 1. Compared with other systems, **ZnSm–X** exhibits superior sensitivity across a wide temperature range, demonstrating excellent room‐temperature sensing performance and promising application potential.

**TABLE 1:** Comparison of the relative sensitivity of molecular complexes with proportional and temperature measurement characteristics.

**Compounds**	**Work temperature**	**Relative sensitivity**	**Refs**.
**SmAu**	10–300 K	3.5% K^−1^ (at 80 K)	[25]
**Tb**_**0.98**_ **Eu**_**0.02**_ **TPDB**	291–321 K	7.32% K^−1^ (at 321 K)	[26]
**cycEu‐phTb**	10–200 K	1.86% K^−1^ (at 200 K)	[27]
{**YbCo**_**2**_}	10–300 K	4.6% K^−1^ (at 50 K)	[28]
**FπVC**	173–303 K	3.0% K^−1^ (at 253 K)	[29]
**HoCo**	10–300 K	6.9% K^−1^ (at 30 K)	[30]
**ZnSm‐X**	233–333 K	12.14% K^−1^ (at 268 K)	This work

## 2 Results and Discussion

### 2.1 Preparation of Single Crystals

**FIGURE 1:**
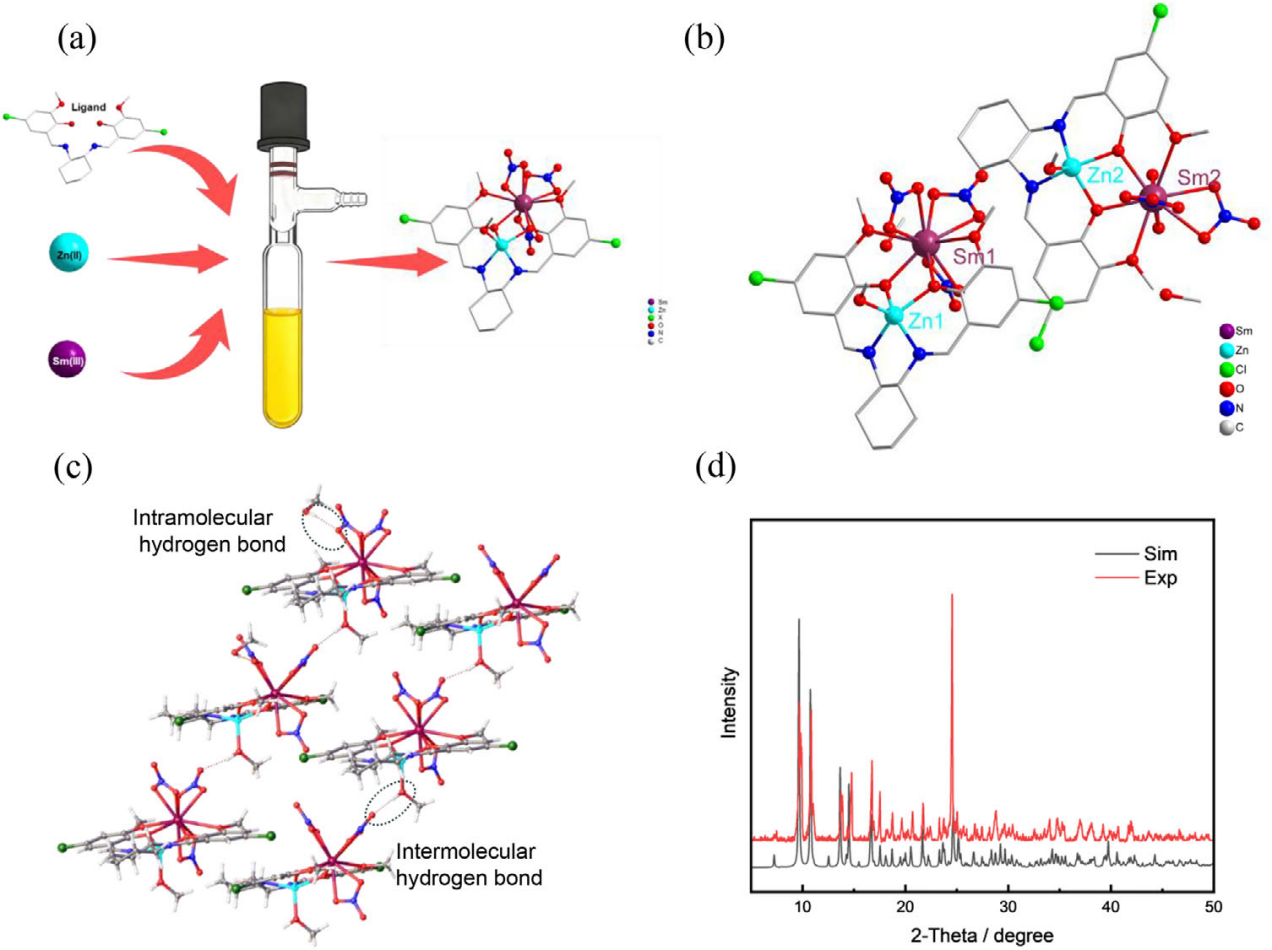
Molecular structure of **ZnSm‐X**. (a) Scheme of one‐pot synthesis. (b) Crystal structure diagram. (c) Schematic diagram of the packing arrangement along one crystallographic axis and hydrogen bonding. (d) Experimental and calculated XRD patterns of **ZnSm‐Cl**.

Halogenated Schiff base ligands *R*, *R*‐**H**_**2**_ **L** (**H**_**2**_ **L** = (N, N'‐bis(3‐methoxy‐5‐X‐salicylidene)cyclohexane‐1,2‐diamine)) (Scheme S1), Zn(OAc)_2_·2H_2_O, and Sm(NO_3_)_3_·6H_2_O were dissolved in a mixed solvent of methanol and acetonitrile, and a one‐pot reaction was heated to 70°C for four hours, yielding **ZnSm** crystals on the walls of the reaction vessel (Figure 1a). From the nuclear magnetic resonance (NMR) data, it can be seen that the ligand is free of impurities (Figure S1). Single‐crystal diffraction analysis at 180 K indicated that the **ZnSm‐Cl** crystal crystallized in the P2_1_ polar space group. The asymmetric unit consists of two crystallographically independent heterometallic complexes (Zn1…Sm1 and Zn2…Sm2). In the complex, phenolate bridges connect Zn^2+^ and Sm^3+^ ions, with Zn1…Sm1 and Zn2…Sm2 distances of 3.4948(4) Å and 3.4907(4) Å, respectively (Figure 1b). The Zn^2+^ cation is located in the N_2_O_2_ pocket of the Schiff base and a methanol molecule, forming a five‐coordinate pyramidal geometry. The methanol molecule acts as a hydrogen‐bond donor connecting to the nitrate and forming an intermolecular hydrogen bond with a bond length of 1.902(5) Å. This interaction plays a crucial role in crystal growth and stacking. The Sm^3+^ ion coordinates with external phenoxide/methoxy O, O'‐donor atoms, while three nitrate ions occupy the remaining coordination sites of Sm^3+^. An intermolecular nitrate ion also forms a hydrogen bond with a methanol molecule, with a bond length of 2.007(5) Å (Figure 1c). The stacking of **ZnSm‐Cl** molecules along the *a*, *b*, and *c* axes is shown for better visualization (Figure S2). Analysis of the single‐crystal structure data (Table S1) indicates that, there are two crystallographically inequivalent molecules **ZnSm‐X**, the distance between Zn^2+^ and Sm^3+^ ions is calculated using the average distance (***d***), as shown in Table 2. As the radius of the halogen atom increases, ***d*** increases correspondingly, indicating that **ZnSm‐Cl**, with its smaller ***d***, exhibits greater structural rigidity. Compared to the intermolecular distance of **ZnSm**, the halogen‐substituted intermolecular distances are all less than 3.53109(4)Å. In the powder XRD patterns of the samples, the experimentally measured powder XRD of single‐crystal **ZnSm‐Cl** shows characteristic peaks highly consistent with the simulated XRD results, confirming the high purity of **ZnSm‐Cl**. Additionally, the powder XRD patterns of **ZnSm**, **ZnSm‐Br**, and **ZnSm‐I** also exhibit excellent agreement with the simulated results (Figure S3).

**TABLE 2:** Intermolecular spacing of ZnSm‐X and ZnSm.

**Complex**	***d* (Å)**
**ZnSm‐Cl**	3.493 (4)
**ZnSm‐Br**	3.502 (4)
**ZnSm‐I**	3.513 (4)
**ZnSm**	3.531 (4)

### 2.2 Relative Sensitivity of ZnSm‐Cl

**FIGURE 2:**
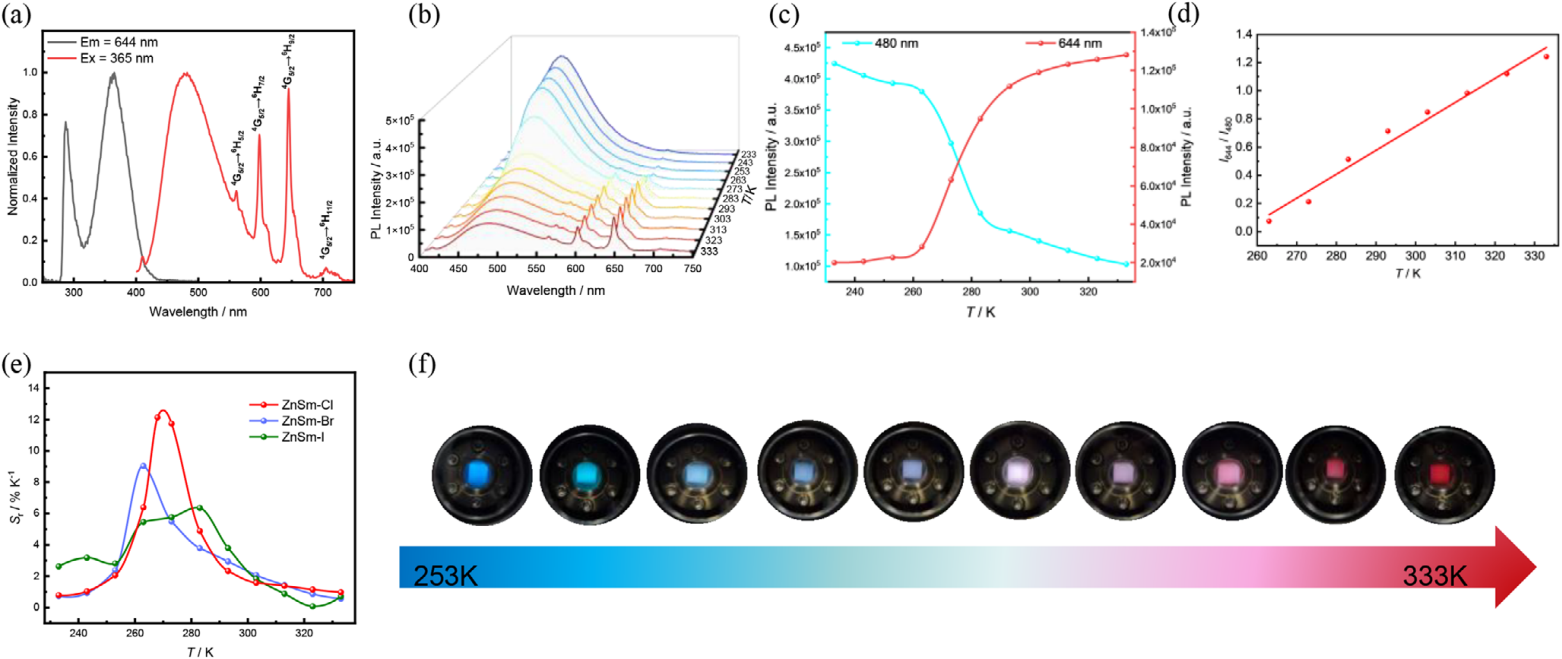
Temperature dependence of emission properties for **ZnSm‐Cl**. (a) Excitation and emission spectra of **ZnSm‐Cl**. (b) Emission spectra recorded between 233 K and 333 K in toluene solutions (1.0 × 10^−5^ m) (excitation at 365 nm). (c) Temperature dependence of the integrated intensities at 480 nm and 644 nm. (d) Temperature dependence of the intensity ratio (*I*_644_/*I*_480_), the result fitted using Equation (1). (e) Measured results of the temperature‐dependent relative sensitivity, the result calculated using Equation (2). (f) The luminescent color of **ZnSm‐Cl** at different temperatures (253–333 K).

To explore the potential of **ZnSm‐X** as a ratiometric thermometer, we further investigated its temperature‐dependent photoluminescence properties. We measured the emission spectrum of **ZnSm‐Cl** in solution at room temperature and found that it exhibits dual emission characteristics. The emission peak at 480 nm may correspond to the luminescent feature of **ZnL** [31], while the emission peaks at 560 nm, 599 nm, and 644 nm are attributed to the characteristic transitions of Sm^3+^ (^4^G_5/2_→^6^H_*J*_, *J* = 5/2, 7/2, 9/2) [32]. According to the excitation spectrum of **ZnSm‐Cl** (Figure 2a), the effective excitation wavelength range for the emission peak of Sm^3+^ at 644 nm is 280–365 nm (as indicated by the black curve), with the optimal excitation wavelength being 365 nm. Figure 2b shows the emission spectra of **ZnSm‐Cl** as a function of temperature. Due to the thermal activation of non‐radiative decay pathways, the emission intensity of **ZnL** at 480 nm decreases significantly with increasing temperature. In contrast, the luminescence emission of Sm^3+^ increases with temperature in the range of 233–333 K, with the emission intensity at 644 nm becoming stronger as the temperature rises. **ZnSm**, **ZnSm‐Br**, and **ZnSm‐I** exhibit **ZnL** emission peaks at 470 nm (Figure S4a), 470 nm (Figure S5a), and 475 nm (Figure S6a), respectively. **ZnSm**, **ZnSm‐Br**, and **ZnSm‐I** show the same variation trend in emission intensity at **ZnL** and Sm^3+^ as **ZnSm‐Cl**. Therefore, by using two different luminescent centers with complementary temperature‐dependent behaviors, ratiometric temperature sensing can be realized. This is because **ZnSm‐X** predominantly emits from **ZnL** at low temperatures, while being dominated by Sm^3+^ at high temperatures, with distinct blue and red color changes. In this system, the temperature‐dependent photoluminescence behavior is due to the negative quenching effect [33, 34]. In the temperature range of 233–333 K (near room temperature), the intensity‐change trends of the two emissions are complementary, which demonstrates the potential of **ZnSm‐X** as a ratiometric thermometer.

Next, we performed a linear fit and analysis of the relationship between the luminescence intensity of the **ZnSm‐Cl** complex and temperature. The photoluminescence behavior of **ZnSm ‐ Cl** may result from energy transfer from the **ZnL** to the Sm^3+^ ion. In addition, we compared the temperature‐dependent luminescence intensities of **ZnSm‐Cl**, **ZnSm** (Figure S4b), **ZnSm‐Br** (Figure S5b), and **ZnSm‐I** (Figure S6b). Figure S4b shows that as the temperature rises, although the photoluminescence of **ZnL** at 470 nm corresponding to **ZnSm **gengrally decreases, an anomalous increase is observed at 293 K. This causes the luminescence intensity ratio *I*_644_/*I*_470_ of the two emission centers to lack a good linear correlation(Figure S4c). Moreover, the biexponential fitting of the luminescence lifetime of 470 nm indicated a luminescence lifetime of 5.444 µs (Figure S4d), whereas typical fluorescent materials have lifetimes in the nanosecond range. Furthermore, based on the low‐temperature phosphorescence tests of **ZnGd‐X** (X = H, Cl, Br, I) mentioned later, the phosphorescence emission of the **ZnL** is around 520 nm. This rules out the possibility that the **ZnL** in **ZnSm** is responsible for the phosphorescence emission. Therefore, it can be concluded that ZnSm exhibits delayed fluorescence characteristics. After excitation, the molecule in the triplet state undergoes reverse intersystem crossing (RISC) to the singlet S_1_ state, followed by radiative decay to the ground state, resulting in a prolonged fluorescence lifetime. In the **ZnSm** sample, the photoexcited energy competes between the RISC process from the T_1_ state to the S_1_ state of **ZnL** and the lumine scence emission of Sm^3+^ at 644 nm (Figure S4e) [35]. Consequently, we did not perform temperature‐dependent photoluminescence testing on the **ZnSm** molecule. In **ZnSm‐Cl**, the **ZnL** emission intensity decreased by nearly 76.5%, while the Sm^3+^ emission at 644 nm increased by approximately 650% within the 233–333 K range (Figure 2c). Sensitivity is an important parameter for temperature sensors, and the appropriate definition of the sensitivity metric allows the evaluation and comparison of thermometers. To enable quantitative comparison of thermometers operating via different mechanisms, the relative sensitivity (*S*_r_) is usually employed [36]. The relative sensitivity needs to be fitted to a linear relationship using the ratio of emission intensities from different emitters in a dual‐emitter system. Therefore, the ratio of the ^4^G_5/2_ → ^6^H_9/2_ (Sm^3+^, 644 nm) transition and the emission intensities of **ZnL** (480 nm) (*I*_644_/*I*_480_) was plotted against temperature, showing a good linear relationship (Figure 2d). This relationship can be expressed by Equation (1).

(1) $$I_{644}/\;I_{480}=0.01697T-4.34432$$

The correlation coefficient reached 0.9794, where *I*_644_ and *I*_480_ represent the luminescence intensities of Sm^3+^ and **ZnL**, respectively, and *T* is the temperature. Similarly, we also performed linear fitting on the emission intensities of **ZnSm‐Br** and **ZnSm‐I**, with correlation coefficients reaching 0.989 (Figure S5c) and 0.967 (Figure S6c), respectively.

For our compounds excited state, the relative sensitivity could be calculated according to the following equation:

(2) $$S_{r}(T)=(\partial (I_{644}/I_{480})/\partial T)/(I_{644}/I_{480})$$

Following Equation (2), the functional relationship between sensitivity and temperature is obtained, as shown in Figure 2e. It can be concluded that **ZnSm‐Cl** maintains high sensitivity in the range of 253–293 K, with its relative sensitivity remaining above 2.06% K^−1^. The maximum relative sensitivity of **ZnSm‐Cl** reaches 12.14% K^−1^ at 268 K, which is among the highest values reported for optical thermometers to date [37]. Similarly, we obtained the maximum *S*_r_ of **ZnSm‐Br** and **ZnSm‐I** to be 9.04% K^−1^ (263 K) and 6.36% K^−1^ (283 K), respectively. Within the 253–293 K range, the relative sensitivities of **ZnSm‐Br** and **ZnSm‐I** remain above 2.39% K^−1^ (Figure S5d) and 2.82% K^−1^ (Figure S6d), respectively.

As shown in Figure 2f, upon exposure to ultraviolet light within the temperature range of 233–333 K, the compound's luminescent color gradually changes from blue to pink and eventually to red. This noticeable color change, visible to the naked eye, greatly enhances the convenience and practicality of the **ZnSm‐Cl** molecular ratiometric thermometer.

### 2.3 Low‐Temperature Phosphorescence and Energy Level Matching Mechanism of ZnSm‐Cl

**FIGURE 3:**
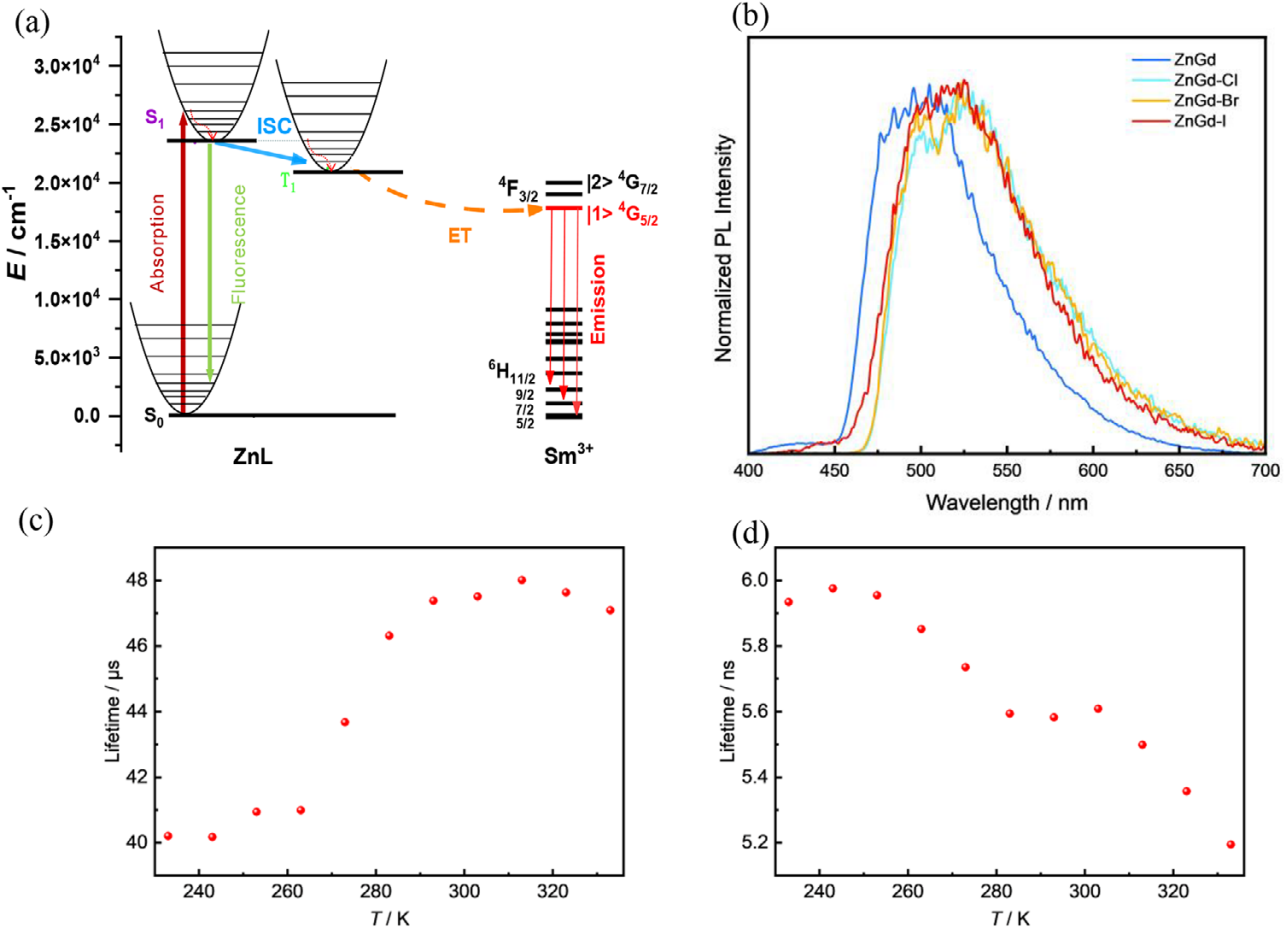
(a) Relative energy levels of **ZnSm‐Cl** and possible energy transfer pathway. (b) Low‐temperature phosphorescence emission of **ZnGd**, **ZnGd‐Cl**, **ZnGd‐Br**, and **ZnGd‐I**. Temperature dependence of the luminescence lifetime of **ZnSm‐Cl**. (c) Sm^3+^ (644 nm) and (d) **ZnL** (480 nm).

During the fluorescence emission process, **ZnSm‐X** employs organic ligands that act as “antennas” to absorb UV excitation energy and transfer it to the rare‐earth ions, and resulting in intense luminescence. According to the Photoinduced Electron Transfer (PET) effect, the imine group, as a donor, can easily transfer an electron to the phenol group, leading to fluorescence quenching. Zn^2+^ is a transition‐metal cation with a tightly bound *d*‐orbital shell that does not induce charge‐separated excited states, thereby restricting energy and electron transfer at the imine site and suppressing the PET effect. Therefore, it facilitates energy transfer from the **ZnL** energy level to Sm^3^^+^ excited state, resulting in promoting luminescence, and the lumine scence variation can be monitored by colorimetric methods (Figure 3a). Since the triplet energy level of **ZnL** is not significantly affected by lanthanide ions, and the lowest excited energy level of Gd^3+^ (^6^P_7/2_→^8^S_7/2_) lies at 32150 cm^−1^, **ZnL** cannot efficiently transfer energy to Gd^3+^ because of its high excited‐state energy. [38] Consequently, it fails to sensitize the characteristic emission of rare‐earth ions. Therefore, the triplet energy level of **ZnL** is determined by the complexes formed with **ZnGd**. The triplet energy level of **ZnL** was obtained through the phosphorescence spectra of **ZnGd** and **ZnGd‐X** in toluene at 77 K (Figure 3b). The single crystal structure data of **ZnGd** and **ZnGd‐X** are shown in Table S2. Among them, the triplet excitation T_1_ energy of **ZnL** in **ZnGd** is 22002 cm^−1^ (Table 3). In **ZnGd‐X**, increasing electronegativity of the halogen substitutions (I, Br, Cl) cause a gradual decrease in the T_1_ energy. The energy difference (Δ*E*) between the corresponding **ZnL** triplet state and Sm^3+^ excited state also decreases accordingly. This precisely confirms the application of the antenna effect, in which the organic ligand transfers energy to the rare earth metal ions, in **ZnSm‐X**. Specifically, the triplet state of Cl‐substituted **ZnL** is closest to the excited state energy level of Sm^3+^, resulting in the highest energy transfer efficiency between the two levels, and the energy transfer efficiency of halogen‐substituted **ZnL** is higher than that of the unsubstituted organic ligand. According to the energy level data of Sm^3+^ ions in the Dieke diagram, the corresponding excited state spectral term ^4^G_5/2_ of Sm^3+^ has a energy of 17825 cm^−1^ [39]. We have illustrated the energy transfer mechanism in Figure 3a, which reveals the intersystem crossing and luminescence emission processes responsible for the dual emission in **ZnSm‐X**. Due to the PET phenomenon, the free ligand emits very weak fluorescence. However, when bound to Zn^2+^ ions, the molecular structure changes from flexible to rigid, resulting in a sharp increase in fluorescence intensity [40]. As the temperature rises, the increased number of T_1_ states promotes the transfer of energy from the T_1_ state of **ZnL** to the excited state of Sm^3+^, resulting in enhanced emission intensity at 644 nm. This energy transfer behavior has been further verified by lifetime measurements. The temperature‐dependent lifetimes of each molecule can be obtained from the biexponential fitting curves of the luminescence lifetime of **ZnSm‐X** (Figure 3c,d; Figure S7). For **ZnSm‐Cl** molecules, the lifetime of the Sm^3+ 4^G_5/2_ → ^6^H_9/2_ (644 nm) transition monitored at 333 K is approximately 46.9 µs, with the luminescence lifetime increasing by 17.5% compared to 233 K. In contrast, the emission lifetime of **ZnL** at 480 nm decreases by nearly 13%, indicating that energy transfer from **Z** **nL** to Sm^3+^ ions may occur within **ZnSm‐Cl**. Similarly, **ZnSm‐Br** and **ZnSm‐I** exhibit the same trend (Figure S8).

**TABLE 3:** Low‐temperature phosphorescence emission of **ZnGd‐X** and **ZnGd**.

Complex	T_1_ (nm)	Energy (cm^−1^)	∆*E* (cm^−1^)
**ZnGd**	454.5	22002	4176
**ZnGd‐Cl**	471.5	21208	3382
**ZnGd‐Br**	469.7	21290	3464
**ZnGd‐I**	463.5	21576	3750
**ZnGd‐NA**	499.6	20014	2189

### 2.4 Antenna Effect of **ZnSm‐X**

**FIGURE 4:**
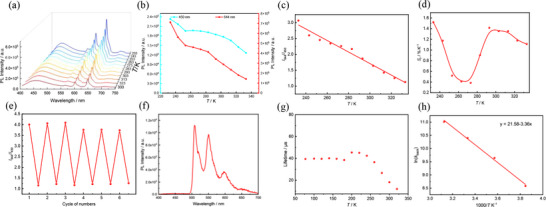
Temperature dependence of emission properties for **ZnSm‐NA**. (a) Emission spectra recorded between 233 and 333 K in toluene solutions (1.0 × 10^−4^ m) (excitation at 365 nm). (b) Temperature dependence of the integrated intensities at 450 nm and 644 nm. (c) Temperature dependence of the intensity ratio (*I*_644_/*I*_450_). (d) Measured results of the temperature‐dependent *S*_r_. (e) The intensity ratio *I*_644_/*I*_450_ exhibited reversibility during thermal cycling between 233 K and 333 K. (f) Low‐temperature phosphorescence emission. (g) Temperature dependence emission lifetime from 77 to 320K. (h) Arrhenius plot of *k*_back_.

Because the T_1_ energy level of halogen‐substituted **ZnL** in the antenna effect happens to be well matched with the Sm^3+^excited state, significant energy transfer from **ZnL** to Sm^3+^ is observed as the temperature increases [41]. For comparison, we preprepared a Schiff base complex with a naphthalene ring skeleton via a one‐pot method, denoted as **ZnSm‐NA**. We then analyzed the relationship between the luminescence intensity of the **ZnSm‐NA** complex and temperature (Figure 4a). It can be seen that as the temperature increases, the emission intensities both **ZnL** emission (at 450nm) and the Sm^3+^ emission (at 644 nm) decrease. This photoluminescence behavior may be caused by back energy transfer (BEnT) from the Sm^3+^ ion to the **ZnL**. Analyzing the relationship between the luminescence intensity of the **ZnSm‐NA** complex and temperature (Figure 4b), the function relationship of the emission intensity ratio of **ZnL** (450 nm) and Sm^3+^ (644 nm) (*I*_644_/*I*_450_) with temperature was obtained through linear fitting (Figure 4c). It was found that while it shows good linearity, the material **ZnSm‐NA** exhibits a pattern opposite to that of the halogenated Schiff base ligands. A minimum point appeared near 273 K (Figure 4d). During the temperature cycle from 233 to 333 K, the *I*_644_/*I*_450_ ratio of the **ZnSm‐NA** sample still shows clear reversibility (Figure 4e). Generally speaking, high sensitization efficiency is achieved only when the energy gap between the excited state of the rare earth ion and the triplet state of the ligand is between 2000 cm^−1^ and 5000 cm^−1^. If the gap is too large, effective energy transfer cannot occur; if it is too small, energy back‐transfer from the excited state of the rare earth ion to the ligand triplet state may take place [42]. Afterward, we used the low‐temperature phosphorescence emission of **ZnGd‐NA** in toluene at 77K to test the energy gap between the naphthalene ‐ based Schiff ‐ baseed ligand and the excited state of Sm^3+^ (Figure 4f). As shown in Table 3, the triplet state T_1_ energy of **ZnGd‐NA** is 20014 cm^−1^, and the energy gap between this ligand and the excited state of Sm^3+^ is 2189 cm^−1^, which is the smallest value and approachs the lower limit of the efficient sensitization range in this system. The excited state of Sm^3+^ readily back‐transfers energy to the triplet state of the antenna ligand, resulting in the quenching of Sm^3+^ luminescence [43]. The naphthalene ‐ based Schiff ‐ based ligand induces splitting of the T_1_ excited state energy level of the **ZnL**, which results in three emission peaks [44]. The temperature dependence of the energy back‐transfer rate is expected to follow an Arrhenius‐type equation with an energy barrier *E*_a_ [45]. To analyze in detail the mechanism of energy back‐transfer from the excited state of Sm^3+^ in **ZnSm‐NA** to the triplet state of **ZnL**, we used kinetic analysis to estimate the energy back‐transfer rate (*k*_back_). It is assumed that *k*_back_ follows the following Arrhenius‐type Equation (3) [46],

(3) $$ln\;k_{back}=ln\left(\frac{1}{\tau_{obs}}-\frac{1}{\tau_{77K}}\right)=lnA-\frac{E_{a}}{RT}$$

where *τ*_obs_ is the emission lifetime, *τ*_77K_ is the emission lifetime at 77 K, *A* is the frequency factor, *E*_a_ is the activation energy, *R* is the gas constant, and *T* is the temperature. According to the temperature‐dependent changes in the emission lifetime of **ZnSm‐NA** measured in Figure 4g, a temperature‐dependent kinetic model was fitted using an Arrhenius plot. The activation energy (*E*_a_) was ultimately determined to be 27.94 kJ/mol (Figure 4h). Since 1 kJ/mol = 83.5935 cm^−1^, the theoretical energy gap between the excited state of Sm^3+^ and the triplet state of **ZnL** can be calculated to be 2335 cm^−1^, which is close to the experimental energy gap value (2189 cm^−1^) obtained from low‐temperature phosphorescence emission. The Arrhenius plot showing a linear relationship can prove that back energy transfer has occurred. Therefore, we have identified the reason why **ZnSm‐NA** cannot achieve good relative sensitivity. In contrast, this conclusion indirectly validates the mechanism by which **ZnSm‐Cl** can achieve good relative stability. On one hand, the singlet‐triplet energy gap of **ZnL** is within an appropriate range, avoiding the impact of RISC on energy transfer. At the same time, the energy gap between the triplet state of **ZnL** and the excited state of Sm^3+^ is also within an appropriate range, preventing energy back‐transfer that could quench the luminescence of rare‐earth ions. On the other hand, phonon‐assisted radiative transitions caused by negative quenching result in **ZnSm‐Cl** exhibiting temperature‐dependent photoluminescence performance with relative sensitivity (*S*_r_) as high as 12.14% at 268K.

### 2.5 Reversibility of the ZnSm‐X Temperature Sensor

**FIGURE 5:**
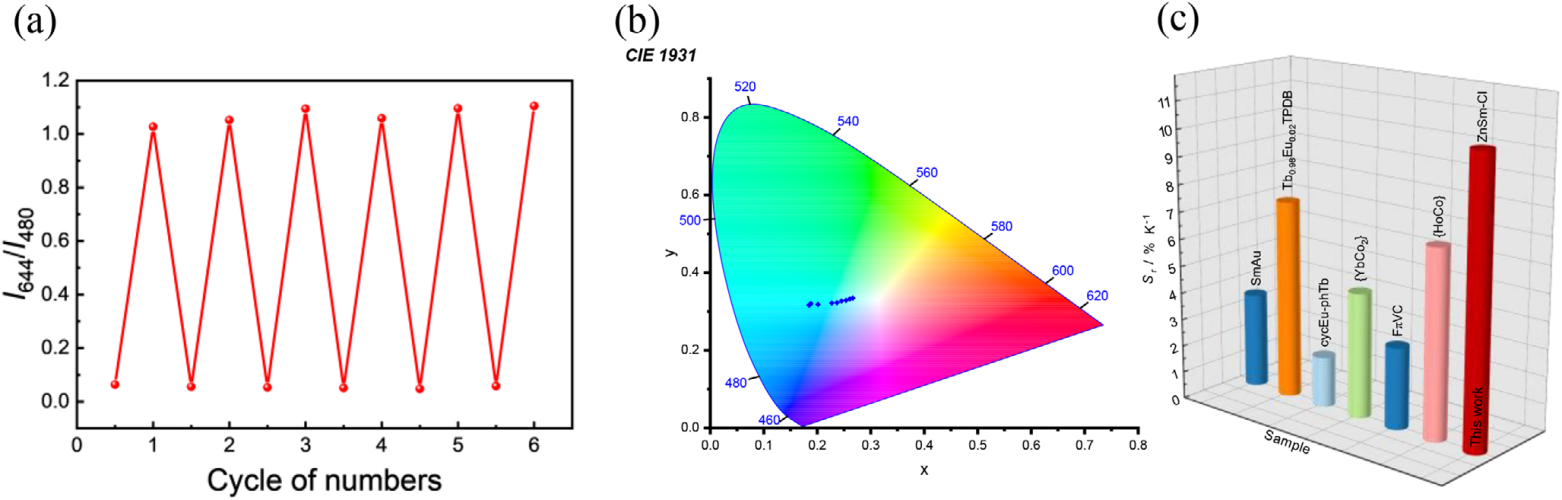
Performance of **ZnSm ‑ Cl** as a ratiometric thermometer.(a) The reversibility of *I*_644_/*I*_480_ was observed during temperature cycling between 233 and 333 K. (b) CIE coordinates (CIE 1931) of the corrected fluorescence spectra. (c) Comparison bar chart of the relative sensitivity of other dual‐core molecular ratiometric thermometers.

To further verify the reversibility of the **ZnSm‐X** temperature sensor, we conducted six consecutive cycles of temperature‐dependent emission tests in the range of 233–333 K (Figure 5a). The dual‐emission intensity ratios at the same temperature in different cycles remained nearly constant, fully demonstrating the excellent reversibility of this system. Figure 5b shows the changes in the International Commission on Illumination (CIE 1931) coordinates corresponding to the **ZnSm‐Cl** calibrated fluorescence spectra. During the range from 233K (chromaticity coordinates x = 0.18, y = 0.32) to 333K (x = 0.27, y = 0.33), significant continuous color changes were observed. The corresponding CIE 1931 chromaticity coordinates of **ZnSm‐Br** and **ZnSm‐I** calibrated fluorescence spectra exhibited the same trend (Figure S9). For **ZnSm‐Br**, the change was from 233K (x = 0.25, y = 0.25) to 333K (x = 0.52, y = 0.35), and for **ZnSm‐I**, from 233K (x = 0.37, y = 0.33) to 333K (x = 0.59, y = 0.36). At low temperatures, the energy transfer from the triplet state of the ligand to the excited state of Sm^3+^ is suppressed, resulting in a lower red emission intensity compared to the blue emission. These unique properties make **ZnSm ‐ X** ideal candidate materials for self‐calibrating optical thermometers. Compared with the relative sensitivity of other latest molecular ratiometric thermometers, the **ZnSm‐Cl** in this work is higher than other molecular complexes, reaching one of the most advanced levels internationally (Figure 5c).

## 3 Conclusion

In conclusion, we have developed **ZnSm‐X** (X = Cl, Br, I, NA) as dual‐emission center molecular materials within a non‐centrosymmetric crystal system, which can serve as a ratiometric molecular thermometer around room temperature. Its fluorescence properties are highly sensitive and tunable with temperature within the range of 253–333 K (at 80 K range) near room temperature. The **ZnL** emits indigo fluorescence around 470 nm, while Sm^3+^ emits orange‐red luminescence at 561 nm, 599 nm, 644 nm, and 710 nm through specific transitions, with their fluorescence changing complementarily with temperature. That is, at low temperatures, **ZnL** emission dominates, and as the temperature rises, Sm^3^^+^ emission becomes dominant. High‐sensitivity, reversible, and color‐distinct ratiometric temperature monitoring is achieved via the antenna effect of energy transfer from **ZnL** to rare earth ions, attributed to thermally induced energy transfer facilitated by energy level matching. Among them, the chlorine‐substituted complex **ZnSm‐Cl** reaches a relative sensitivity of 12.14% K^−1^ at 268 K. Compared with most existing systems, **ZnSm‐X** exhibit superior sensitivity over a wide temperature range, excellent room‐temperature sensing performance, and good application potential, providing an effective approach for research on ratiometric molecular complexes for temperature sensing.

[CCDC 2496633 (ZnSm); CCDC 2496626 (ZnSm‐Cl); CCDC 2496632 (ZnSm‐Br); CCDC 2496627 (ZnSm‐I); CCDC 2496628 (ZnGd); CCDC 2496629 (ZnGd‐Cl); CCDC 2496630 (ZnGd‐Br); CCDC 2496631 (ZnGd‐I); CCDC 2518294 (ZnSm‐NA); CCDC 2518295 (ZnGd‐NA)] contain the supplementary crystallographic data for this paper. These data can be obtained free of charge from The Cambridge Crystallographic Data Centre via www.ccdc.cam.ac.uk/data_request/cif.]

## Funding

This work was supported by the National Natural Science Foundation of China (Grant Nos. 22573003, 22131003).

## Conflicts of Interest

The authors declare no conflict of interest.

## Supporting information




**Supporting File**: advs75323‐sup‐0001‐SuppMat.docx.

## Data Availability

The data that support the findings of this study are available on request from the corresponding author. The data are not publicly available due to privacy or ethical restrictions.
