# Supplementary material for: Antenna Effect in Halogen‐Containing ZnSm Coordination Compounds: Utilizing Colorimetry for a Room‐Temperature Tunable Ratiometric Molecular Thermometer
Source: Adv Sci (Weinh). 2026 Apr 20;13(38):e75323. doi: 10.1002/advs.75323 (PMC13335751; doi:10.1002/advs.75323)
Supplement: Supplementary file 1 — Supporting File: advs75323‐sup‐0001‐SuppMat.docx. [file ADVS-13-e75323-s001.docx]

**Supporting Information**

**Antenna Effect in Halogen-Containing ZnSm Coordination Compounds: Utilizing Colorimetry for a Room-Temperature Tunable Ratiometric Molecular Thermometer**

*Ye Xia,^1,2,§^ Yingshan Xue,^2,3,§^ Feng Pan,^2^ Ai Wang,^5,2^ Hong Huang,^1^ Zhenxing Li,^3^ Bing-Wu Wang,^*,2^ and Song Gao^*,1,2,4^*

^1^Y. Xia, H. Huang, Prof. S. Gao

Spin-X Institute, School of Chemistry and Chemical Engineering, South China University of Technology, Guangzhou 510641, P. R. China. E-mail: gaosong@scut.edu.cn

^2^Y. Xia, Y. Xue, F. Pan, A. Wang, Prof. B. Wang, Prof. S. Gao

Beijing National Laboratory for Molecular Sciences, College of Chemistry and Molecular Engineering, Peking University, Beijing 100871, P. R. China. E-mail: wangbw@pku.edu.cn

^3^Y. Xue, Prof. Z. Li

State Key Laboratory of Heavy Oil Processing, College of New Energy and Materials, China University of Petroleum (Beijing), Beijing 102249, China

*^4^*Guangdong Basic Research Center of Excellence for Functional Molecular Engineering, School of Chemistry, Sun Yat-sen University, Guangzhou 510275, P. R. China.

^5^Prof. A. Wang

Key Laboratory of Chemical Biology and Molecular Engineering of the Education Ministry, Institute of Molecular Science, Shanxi University, Taiyuan, 030006, China

*Corresponding Author.

E-mail: [wangbw@pku.edu.cn](mailto:*wangbw@pku.edu.cn); [gaosong@pku.edu.cn](mailto:*gaosong@scut.edu.cn)

^§^These authors contributed equally to this work.

**KEYWORS** photoluminescence, molecular ratiometric thermometer, rare earth, dinuclear complex, halogen substitution

**Supplementary contents**

**Experimental Section**

Synthesis.

Single-Crystal Structure Determination.

Elemental Analysis.

Powder XRD Analysis.

Fluorescence spectrum.

Supporting figures.

Supporting tables.

References.

**Supplementary Figures**

**Figure S1.** Synthesis pathway and Structure of the enantiopure **1*R*, 2*R*-H_2_L**.

**Figure S2.** Molecular packing diagram of **ZnSm-Cl** complex. Along the (a) a-axis, (b) b-axis, and (c) c-axis.

**Figure S3.** The XRD of (a) **1*R*, 2*R*-H_2_L**, (b) **1*R*, 2*R*-H_2_L-Cl**, (c) **1*R*, 2*R*-H_2_L-Br**, (d) **1*R*, 2*R*-H_2_L-I**.

**Figure S4.** Temperature dependence of emission properties for **ZnSm**. (a) Emission spectra recorded between 233 and 333 K in toluene solutions (1.0 × 10^–4^ M) (excitation at 365 nm). (b) Temperature dependence of the integrated intensities at 470 nm and 644 nm. (c) Fluorescence lifetime curve at the 470 nm emission peak.

**Figure S5.** Temperature dependence of emission properties for **ZnSm-Br**. (a) Emission spectra recorded between 233 and 333 K in toluene solutions (1.0 × 10^–4^ M) (excitation at 365 nm). (b) Temperature dependence of the integrated intensities at 470 nm and 644 nm. (c) Temperature dependence of the intensity ratio (*I*_644_/*I*_470_). (d) Measured results of the temperature-dependent *S*_r_.

**Figure S6.** Temperature dependence of emission properties for **ZnSm-I**. (a) Emission spectra recorded between 233 and 333 K in toluene solutions (1.0 × 10^–4^ M) (excitation at 365 nm). (b) Temperature dependence of the integrated intensities at 475 nm and 644 nm. (c) Temperature dependence of the intensity ratio (*I*_644_/*I*_475_). (d) Measured results of the temperature-dependent *S*_r_.

**Figure S7.** Temperature dependence of the luminescence lifetime of **ZnSm-X**. Temperature dependence of the luminescence lifetime of Sm^3+^ (644 nm) (a) **ZnSm-Cl**, (b) **ZnSm-Br**, and (c) **ZnSm-I** in 233K ~ 333K. Temperature dependence of the luminescence lifetime of **ZnL** (d) **ZnSm-Cl**, (e) **ZnSm-Br**, and (f) **ZnSm-I** in 233K ~ 333K.

**Figure S8.** Temperature dependence of the luminescence lifetime of **ZnSm-Br**. (a) Sm^3+^ (644 nm) and (b) **ZnL** (470 nm). Temperature dependence of the luminescence lifetime of **ZnSm-I**. (c) Sm^3+^ (644 nm) and (d) **ZnL** (475 nm).

**Figure S9.** CIE coordinates (CIE 1931) of the corrected fluorescence spectra of (a) **ZnSm-Cl** and (b) **ZnSm-I**.

**Supplementary Tables**

**Table S1**. Crystal data and structure refinement for 1*R*,2*R*-ZnLSm(CH_3_OH)(NO_3_)_3_.

**Table S2**. Crystal data and structure refinement for 1*R*,2*R*-ZnLGd(CH_3_OH)(NO_3_)_3_.

**Experimental Section**

**Synthesis.**

All experiments were carried out under aerobic conditions. All the solvents in these experiments were analytical grade. The lanthanide nitrate salts were purchased from Energy Chemical. (1*R*,2*R*) -(+)-1,2-Diphenylethylenediamine was purchased from Energy Chemical. The ligand Phenol, 2,2'-[[(1*R*,2*R*)-1,2-diphenyl-1,2-ethanediyl] bis[(*E*)-nitrilomethylidyne]] has been synthesized according to a well-established procedure from the literature.[1] The [1*R*,2*R*-ZnLSm(CH_3_OH)(NO_3_)_3_] (**ZnSm-X**) crystals were grown using the solvothermal method. Dissolve 1*R*, 2*R*-H_2_L (0.06 mmol, 0.0327 g), Zn(OAc)_2_·2H_2_O (0.06 mmol, 0.0131 g), and Sm(NO_3_)_3_·6H_2_O (0.06 mmol, 0.0266 g) in 5 mL of methanol. Heating to 75 ℃ overnight in a sealed tube formed crystals on the sealed rock wall. Obtain colorless, large-sized single crystals of 1*R*,2*R*-ZnLSm(CH_3_OH)(NO_3_)_3_. Elemental analysis calcd for **ZnSm** (exp.): C 34.58 (35.06), H 3.668 (3.583), N 6.93 (7.113). Elemental analysis calcd for **ZnSm-Cl** (exp.): C 30.98 (31.49), H 3.21 (3.304), N 7.27 (7.6523). Elemental analysis calcd for **ZnSm-Br** (exp.): C 28.77 (28.71), H 2.799 (3.011), N 6.93 (6.975). Elemental analysis calcd for **ZnSm-I** (exp.): C 25.51 (26.25), H 2.754 (2.754), N 6.08 (6.37). **ZnSm-NA** is synthesized in a one-pot reaction, in which the original halogenated phenyl ring of 1R,2R-H2L is replaced by a 2-hydroxy-3-methoxy-1-naphthaldehyde moiety.[2] Elemental analysis calcd for **ZnSm-NA** (exp.): C 41.56 (41.87), H 3.38 (3.587), N 5.96 (5.86). The [1*R*,2*R*-ZnLGd(OAc)(NO_3_)_2_] complex was precipitated using the solvothermal method. Dissolve 1*R*, 2*R*-H_2_L (0.06 mmol, 0.0327 g), Zn(OAc)_2_·2H_2_O (0.06 mmol, 0.0131 g), and Gd(NO_3_)_3_·6H_2_O (0.06 mmol, 0.0270 g) in 5 mL of methanol. Heating to 75 ℃ was performed for 6 hours. Obtain colorless, large-size single crystals of 1*R*,2*R*-ZnLGd(OAc)(NO_3_)_2_ by ether gas diffusion methods.

CCDC 2496633 (**ZnSm**); CCDC 2496626 (**ZnSm-Cl**); CCDC 2496632 (**ZnSm-Br**); CCDC 2496627 (**ZnSm-I**); CCDC 2496628 (**ZnGd**); CCDC 2496629 (**ZnGd-Cl**); CCDC 2496630 (**ZnGd-Br**); CCDC 2496631 (**ZnGd-I**); CCDC 2518294 (**ZnSm-NA**); CCDC 2518295 (**ZnGd-NA**). These are the crystallographic data accompanying this article. These data can be obtained free of charge from The Cambridge Crystallographic Data Centre.

**Single-Crystal Structure Determination.**

A colorless 1*R*,2*R*-ZnLSm(OAc)(NO_3_)_2_ crystal (0.33 × 0.29 × 0.06 mm^3^) was selected using an optical microscope for single-crystal XRD analysis. The diffraction data were collected by using graphite-monochromatized Mo Kα radiation (*λ* = 0.71073 Å) at 180 (2) K on an Agilent Bruker D8. The collection of the intensity data, cell refinement, and data reduction were carried out with the program CrysAlisPro. The structure was solved by the direct method with program SHELXS and refined with the least-squares program SHELXL. Final refinements include anisotropic displacement parameters. The structure was verified using the ADDSYM algorithm from the program PLATON,^[4]^ and no higher symmetry was found. Details of crystal parameters, data collection, and structure refinement are summarized in Table S1.

**Elemental Analysis.**

Elemental analyses for C, H, N were performed on an EA1110 microelemental analyzer. confirmed the presence of C, H and N.

**Powder XRD Analysis.**

Powder X-ray diffraction measurements of the 1*R*,2*R*-ZnLSm(CH_3_OH)(NO_3_)_3_ sample was carried out with a Miniflex 600 diffractometer equipped with an incident beam monochromator set for Cu Kα radiation (*λ* = 1.5418 Å). The 2*θ* range was 5-50° with a scan step width of 0.02° and a fixed counting time of 0.20 s/step.

**Fluorescence spectrum**

The emission and excitation spectra for the samples were recorded by a Horiba FluoroMax fluorescence spectrometer. The temperature-dependent emission spectra for the powders of **ZnSm-X** in Toluene solution were recorded by a Horiba FluoroMax fluorescence spectrometer with a OXFORD Temperature Control Solution OPTISTAT-CFS. The luminescence decay curve was recorded on an Edinburgh Instrument FLS980 spectrometer. The luminescence decay curve of 1*R*,2*R*-ZnLSm(CH_3_OH)(NO_3_)_3_ was measured using a 365 nm laser as the light source.

**Supplementary Figures.**


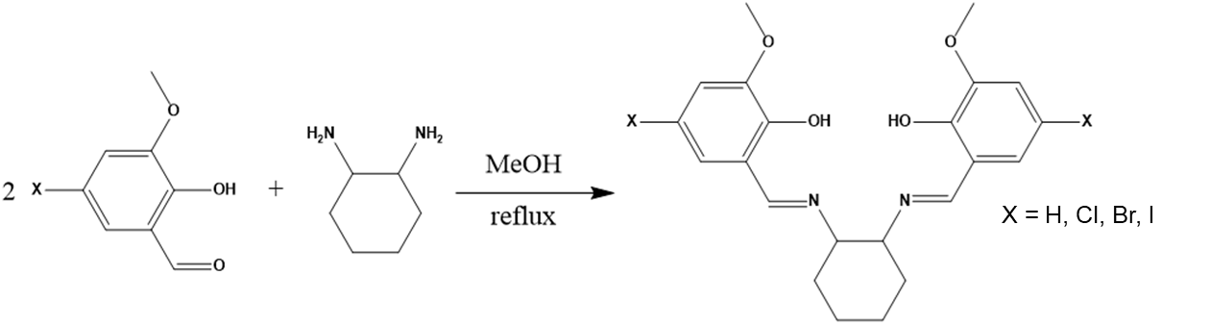


**Scheme S1.** Synthesis pathway and Structure of the enantiopure **1*R*, 2*R*-H_2_L**.


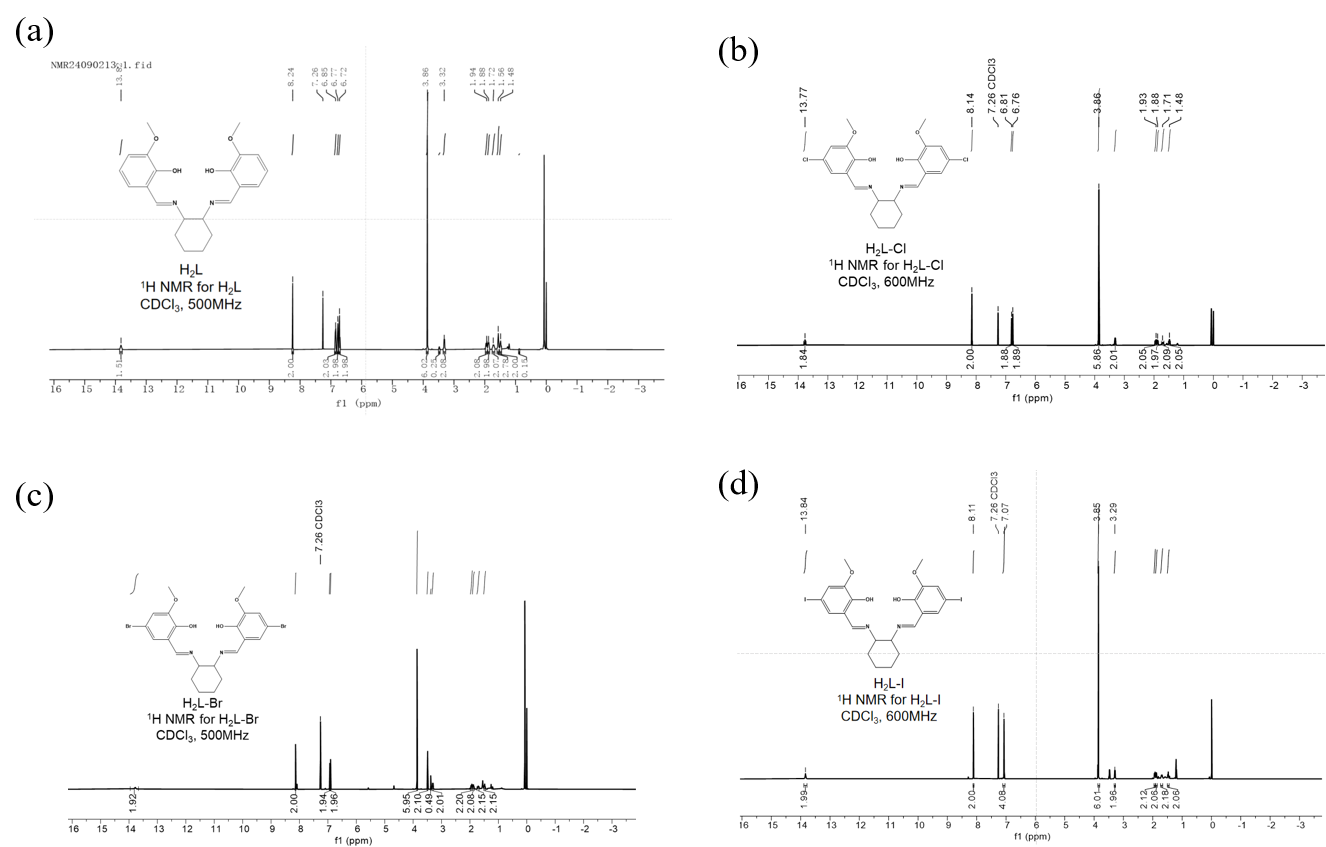


**Figure S1.** The Schiff base ligands are labeled according to different ligands, and the coordination at the benzene ring before and after halogenation is -H, -Cl, -Br, -I, respectively. Molecular structure and Nuclear Magnetic Resonance (NMR) of the enantiopure (a) **1*R*, 2*R*-H_2_L-H**, (b) **1*R*, 2*R*-H_2_L**-Cl, (c) **1*R*, 2*R*-H_2_L**-Br, (d) **1*R*, 2*R*-H_2_L-**I.


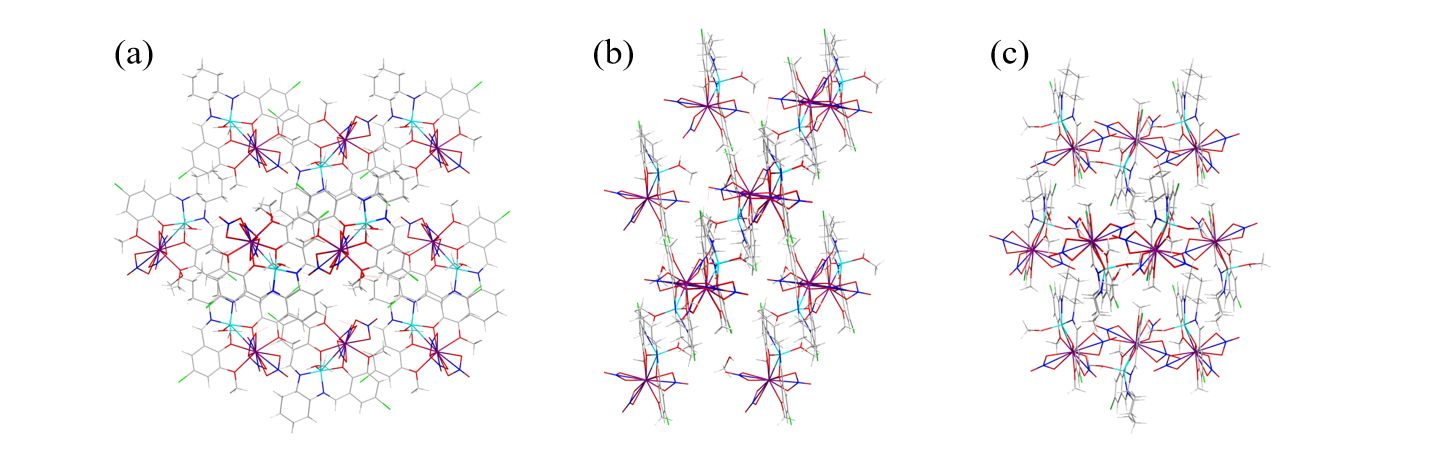


**Figure S2.** Molecular packing diagram of ZnSm-Cl complex. (a) Along the a-axis. (b) Along the b-axis. (c) Along the c-axis.


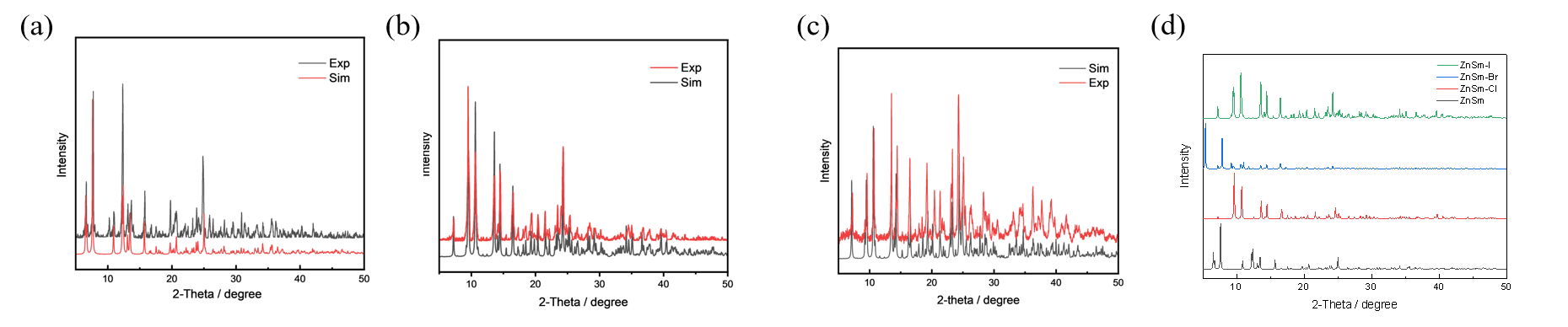


**Figure S3.**  Experimental and calculated XRD patterns of (a) **ZnSm**, (b) **ZnSm-Br**, (c) **ZnSm-I.** (d) XRD comparison chart of four molecules.

**Figure S4.** Temperature dependence of emission properties for **ZnSm**. (a) Emission spectra recorded between 233 and 333 K in toluene solutions (1.0 × 10^–4^ M) (excitation at 365 nm). (b) Temperature dependence of the integrated intensities at 470 nm and 644 nm. (c) Temperature dependence of the intensity ratio (*I*_644_/*I*_470_). (d) Luminescence lifetime curve at the 470 nm emission peak in 297K. (e) Relative energy levels and the energy transfer pathways of delayed fluorescence.


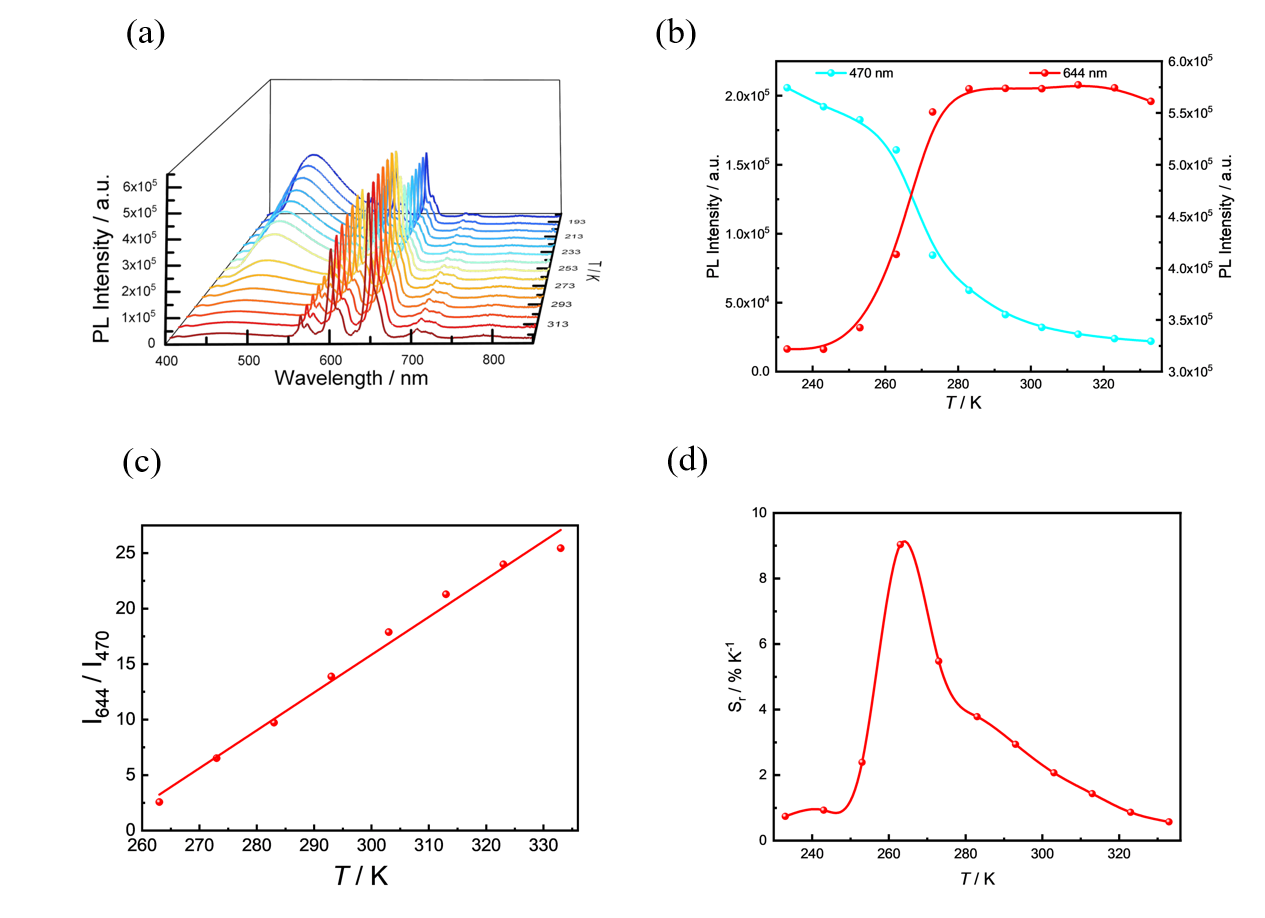


Figure S5. Temperature dependence of emission properties for ZnSm-Br. (a) Emission spectra recorded between 233 and 333 K in toluene solutions (1.0 × 10^–4^ M) (excitation at 365 nm). (b) Temperature dependence of the integrated intensities at 470 nm and 644 nm. (c) Temperature dependence of the intensity ratio (*I*_644_/*I*_470_). (d) Measured results of the temperature-dependent *S*_r_.


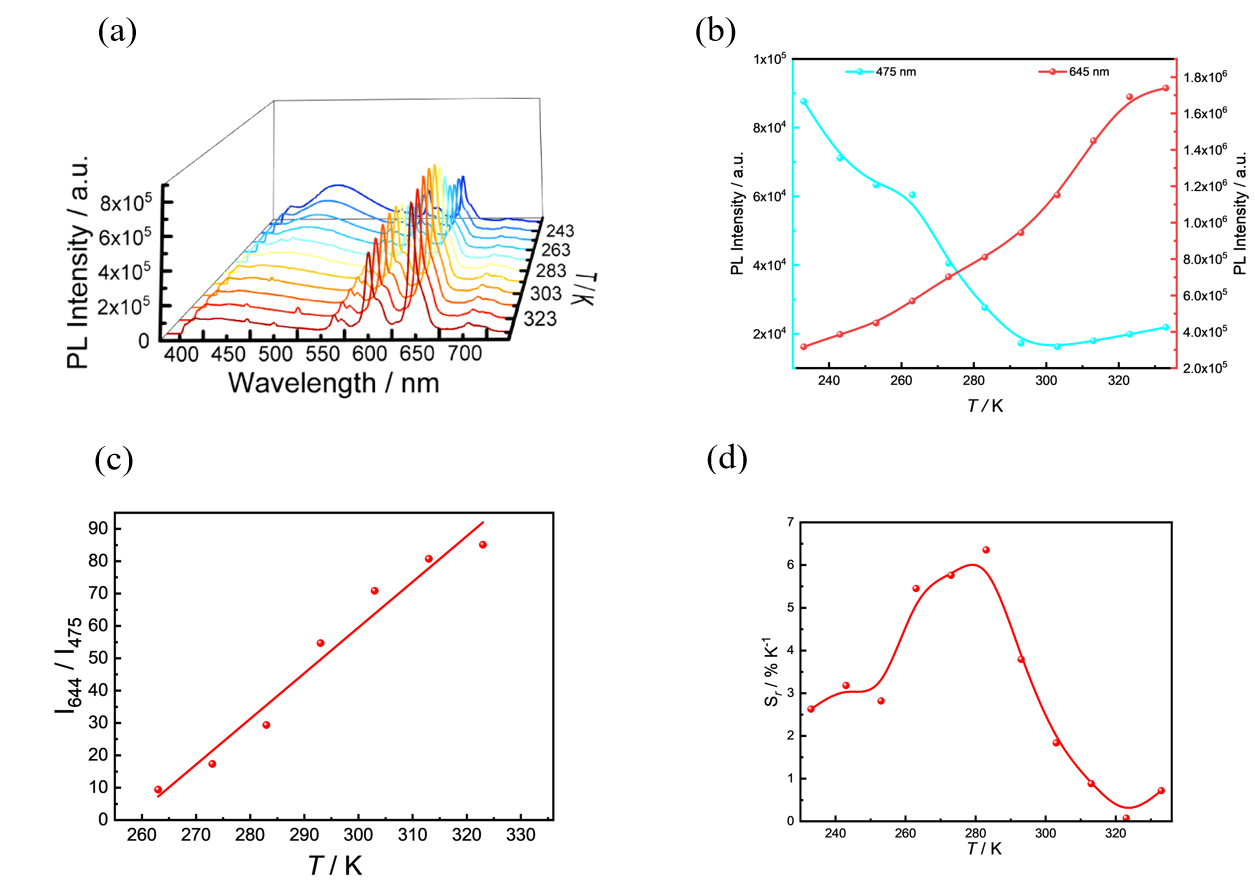


Figure S6. Temperature dependence of emission properties for ZnSm-I. (a) Emission spectra recorded between 233 and 333 K in toluene solutions (1.0 × 10^–4^ M) (excitation at 365 nm). (b) Temperature dependence of the integrated intensities at 475 nm and 644 nm. (c) Temperature dependence of the intensity ratio (*I*_644_/*I*_475_). (d) Measured results of the temperature-dependent *S*_r_.

**Figure S7.** Temperature dependence of the luminescence lifetime of **ZnSm-X**. Temperature dependence of the luminescence lifetime of Sm^3+^ (644 nm) (a) **ZnSm-Cl**, (b) **ZnSm-Br**, (c) **ZnSm-I**, and (d) **ZnSm-NA** in 233K ~ 333K. Temperature dependence of the luminescence lifetime of **ZnL** (e) **ZnSm-Cl**, (f) **ZnSm-Br**, (g) **ZnSm-I**, and (h) **ZnSm-NA** in 233K ~ 333K.


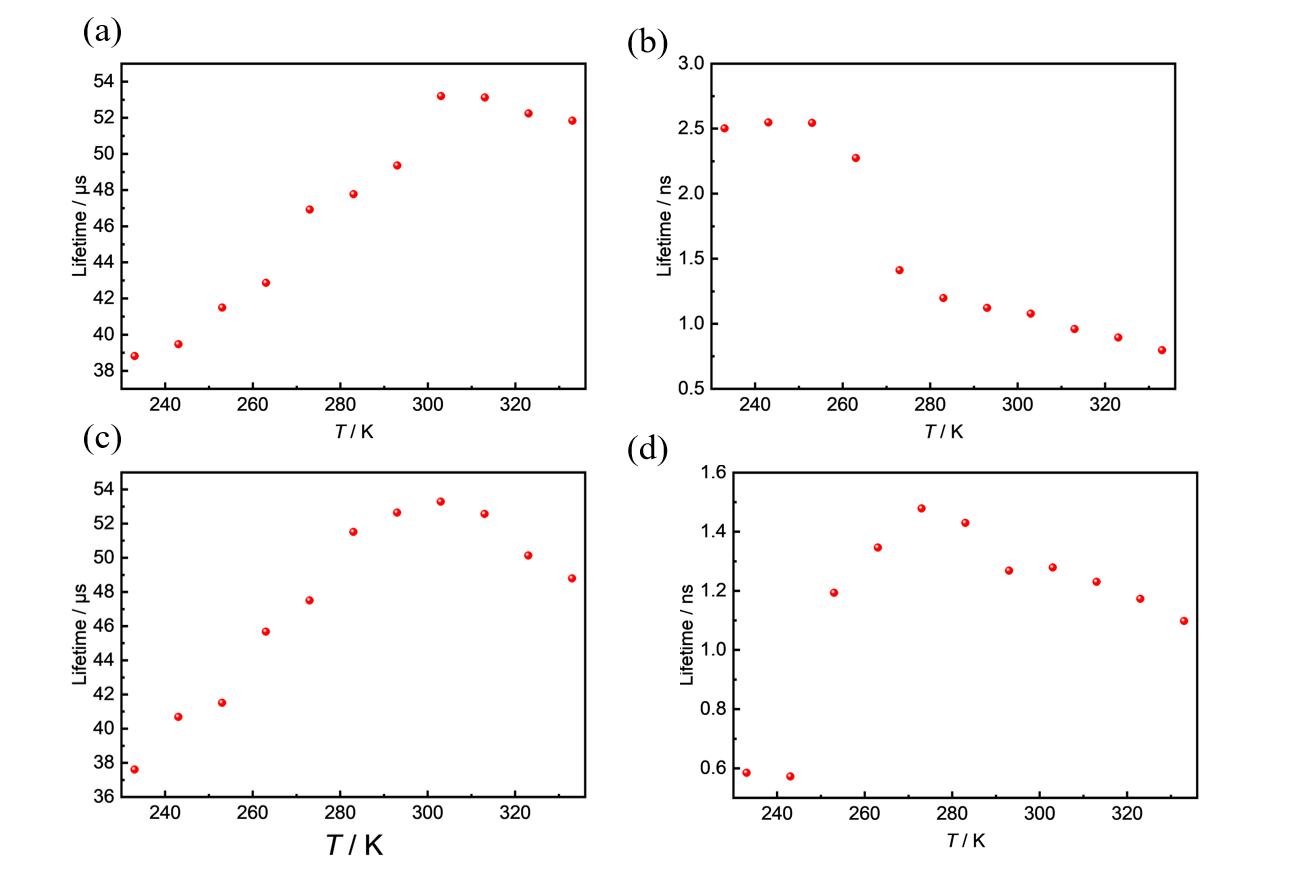


**Figure S8.** Temperature dependence of the luminescence lifetime of **ZnSm-Br**. (a) Sm^3+^ (644 nm) and (b) **ZnL** (470 nm). Temperature dependence of the luminescence lifetime of **ZnSm-I**. (c) Sm^3+^ (644 nm) and (d) **ZnL** (475 nm).


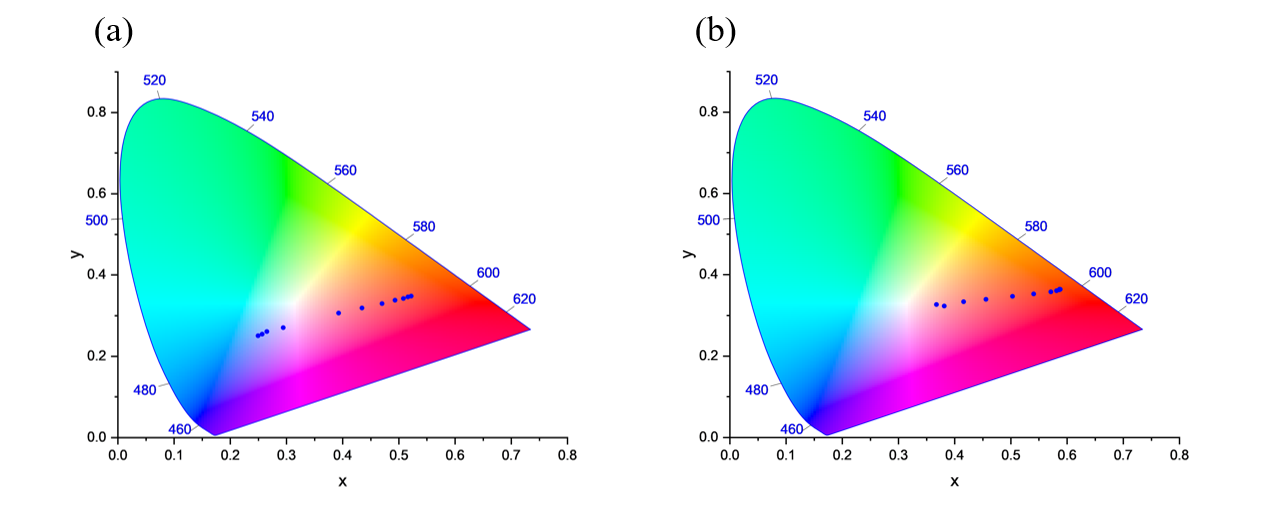


**Figure S9.** CIE coordinates (CIE 1931) of the corrected fluorescence spectra of (a) **ZnSm-Cl** and (b) **ZnSm-I**.

Supporting tables.

Table S1. Crystal data and structure refinement for 1*R*,2*R*-ZnLSm(CH_3_OH)(NO_3_)_3_.

| Crystal data | **ZnSm** | **ZnSm-Cl** | **ZnSm-Br** | **ZnSm-I** | **ZnSm-NA** |
| --- | --- | --- | --- | --- | --- |
| Chemical formula | C_23_H_28_ClN_4_O_11_SmZn·CH_4_O | C_23_H_26_Cl_2_N_5_O_14_  SmZn·CH_4_O | C_23_H_26_Br_2_N_5_O_14_  SmZn·CH_4_O | C_23_H_26_I_2_N_5_O_14_  SmZn·CH_4_O | C_32_H_31_N_4_O_12_SmZn·CHCl_3_ |
| *M*_r_ | 819.70 | 915.15 | 1002.32 | 1098.05 | 998.75 |
| Crystal system, space group | Triclinic, $\text{P}_{\text{1}}^{\text{-}}$ | Monoclinic, *P*2_1_ | Monoclinic, *P*2_1_ | Monoclinic, *P*2_1_ | Monoclinic, P2_1_/*c* |
| Temperature (K) | 180 | 180 | 180 | 180 | 180 |
| *a*, *b*, *c* (Å) | 8.1798 (2), 14.0758 (4), 14.4203 (4) | 11.0779 (2), 18.3215 (3), 16.3602 (3) | 11.2507 (4), 18.3556 (3), 16.4174 (3) | 11.1561 (2), 18.5945 (3), 16.6929 (3) | 11.8000 (3), 13.2177 (3), 27.1887 (7) |
| b (°) | 69.516 (3), 84.411 (2), 82.714 (2) | 90.370 (2) | 90.740 (2) | 90.960 (2) | 100.012 (2) |
| *V* (Å^3^) | 1540.26 (8) | 3320.46 (10) | 3390.13 (15) | 3462.32 (10) | 4176.01 (18) |
| *Z* | 2 | 4 | 2 | 4 | 1 |
| Radiation type | Mo *K*a | Mo *K*a | Mo *K*a | Mo *K*a | Mo *K*a |
| m (mm^-1^) | 2.82 | 2.71 | 4.85 | 4.23 | 2.23 |
| Data collection | | | | | |
| Diffractometer | XtaLAB AFC12 (RINC): Kappa single | | | | |
| Absorption correction | Multi-scan  CrysAlisPro 1.171.42.72a (Rigaku Oxford Diffraction, 2022) Empirical absorption correction using spherical harmonics, implemented in SCALE3 ABSPACK scaling algorithm. | | | | |
| *T*_min_, *T*_max_ | 0.712, 1.000 | 0.854, 1.000 | 0.759, 1.000 | 0.652, 1.000 | 0.738, 1.000 |
| No. of measured, independent and  observed [*I* > 2s(*I*)] reflections | 44033, 8297, 7566 | 67395, 17179, 15897 | 72142, 17248, 14646 | 72395, 17225, 15018 | 71435, 11159, 9130 |
| *R*_int_ | 0.036 | 0.033 | 0.041 | 0.048 | 0.038 |
| (sin q/l)_max_ (Å^-1^) | 0.713 | 0.706 | 0.708 | 0.707 | 0.704 |
| Refinement | | | | | |
| *R*[*F*^2^ > 2s(*F*^2^)], *wR*(*F*^2^), *S* | 0.023, 0.051, 1.12 | 0.029, 0.068, 1.05 | 0.032, 0.063, 1.04 | 0.031, 0.067, 1.03 | 0.041, 0.083 1.08 |
| No. of reflections | 8297 | 17179 | 17248 | 17225 | 11159 |
| No. of parameters | 439 | 874 | 914 | 889 | 509 |
| No. of restraints | 236 | 4 | 61 | 12 | 73 |
| H-atom treatment | H atoms treated by a mixture of independent and constrained refinement | | | | |
| Dρ_max_, Dρ_min_ (e Å^-3^) | 0.56, -0.61 | 1.32, -0.69 | 1.10, -0.71 | 1.27, -1.00 | 1.15, -1.12 |
| Absolute structure parameter | - | 0.010 (5) | 0.010 (5) | 0.018 (11) | - |

Table S2. Crystal data and structure refinement for 1*R*,2*R*-ZnLGd(CH_3_OH)(NO_3_)_3_.

| Crystal data | **ZnGd** | | **ZnGd-Cl** | **ZnGd-Br** | **ZnGd-I** | **ZnGd-NA** |
| --- | --- | --- | --- | --- | --- | --- |
| Chemical formula | C_24_H_27_GdN_4_O_12_Zn·4 CH_4_O | | C_23_H_26_Cl_2_N_5_O_14_SmZn·CH_4_O | C_23_H_26_GdBr_2_N_5_O_14_Zn·CH_4_O | C_23_H_26_GdI_2_N_5_O_14_Zn·CH_4_O | C_32_H_31_GdN_4_O_12_Zn·CHCl_3_ |
| *M*_r_ | 914.28 | | 915.15 | 943.92 | 1103.94 | 1005.64 |
| Crystal system, space group | Monoclinic, *P*2_1_/*n* | | Monoclinic, *P*2_1_ | Triclinic, *P*¯1 | Monoclinic, *P*2_1_ | Monoclinic, *C*2/*c* |
| Temperature (K) | 180 | | 180 | 180 | 180 | 180 |
| *a*, *b*, *c* (Å) | 15.6050 (4), 10.5992 (3), 20.4045 (5) | | 11.0779 (2), 18.3215 (3), 16.3602 (3) | 10.9527 (5), 11.7673 (4), 13.9971 (6) | 11.1993 (5), 18.5844 (7), 16.6027 (6) | 21.6665 (8), 12.7984 (4), 29.4200 (8) |
| b (°) | 103.576 (2) | | 90.370 (2) | 70.366 (4), 74.705 (4), 66.122 (4) | 90.052 (4) | 97.007 (3) |
| *V* (Å^3^) | 3280.62 (15) | | 3320.46 (10) | 1536.49 (13) | 3455.6 (2) | 8097.1 (5) |
| *Z* | 4 | | 4 | 2 | 4 | 8 |
| Radiation type | Mo *K*a | | Mo *K*a | Mo *K*a | Mo *K*a | Mo *K*a |
| m (mm^-1^) | 2.82 | | 2.71 | 5.59 | 4.46 | 2.44 |
| Data collection | | | | | | |
| Diffractometer | | XtaLAB AFC12 (RINC): Kappa single | | | | |
| Absorption correction | Multi-scan  CrysAlisPro 1.171.39.33c (Rigaku Oxford Diffraction, 2017). Empirical absorption correction using spherical harmonics, implemented in SCALE3 ABSPACK scaling algorithm. | | | | | |
| *T*_min_, *T*_max_ | 0.435, 1.000 | | 0.854, 1.000 | 0.733, 1.000 | 0.949, 1.000 | 0.708, 1.000 |
| No. of measured, independent and  observed [*I* > 2s(*I*)] reflections | 67402, 9008, 6436 | | 67395, 17179, 15897 | 25397, 6039, 5545 | 34024, 16097, 13307 | 58864, 10674, 7887 |
| *R*_int_ | 0.060 | | 0.033 | 0.033 | 0.046 | 0.043 |
| (sin q/l)_max_ (Å^-1^) | 0.709 | | 0.706 | 0.617 | 0.708 | 0.698 |
| Refinement | | | | | | |
| *R*[*F*^2^ > 2s(*F*^2^)], *wR*(*F*^2^), *S* | 0.041, 0.095, 1.07 | | 0.029, 0.068, 1.05 | 0.040, 0.087, 1.23 | 0.038, 0.079, 0.99 | 0.042, 0.095, 1.05 |
| No. of reflections | 9008 | | 17179 | 6039 | 16097 | 10674 |
| No. of parameters | 412 | | 874 | 400 | 868 | 484 |
| No. of restraints | 12 | | 4 | 6 | 20 | 31 |
| H-atom treatment | H-atom parameters constrained  *w* = 1/[s^2^(*F*_o_^2^) + (0.0161*P*)^2^ + 12.7414*P*]  where *P* = (*F*_o_^2^ + 2*F*_c_^2^)/3 | | | | | |
| Dρ_max_, Dρ_min_ (e Å^-3^) | 0.98, -0.91 | | 1.32, -0.69 | 1.92, -0.75 | 0.94,-1.09 | 0.55, -0.51 |
| Absolute structure parameter | - | | 0.010 (5) | - | 0.018(15) | - |

# References

[1] Y. Z. Jiang, L. Z. Gong, X. M. Feng, et al., "Salen-Ti(OR)(4) complex catalysed trimethylsilylcyanation of aldehydes," *Tetrahedron* 42 (1997): 14327-14338, https://doi.org/10.1016/s0040-4020(97)00984-8.

[2] E. Sperotto, G. P. M. van Klink, G. van Koten, and J. G. de Vries, "The mechanism of the modified Ullmann reaction," *Dalton Transactions* 43 (2010): 10338-10351, https://doi.org/10.1039/c0dt00674b.
